# Impact of Orchiectomy on Oxidative Stress-Induced Neurodegeneration in the Male Rat Retina: A Proteomic Analysis

**DOI:** 10.3390/antiox15040479

**Published:** 2026-04-12

**Authors:** Khadiza Zaman, Ammar Kapic, Vien Nguyen, Katalin Prokai-Tatrai

**Affiliations:** Department of Pharmacology and Neuroscience, University of North Texas Health Science Center, Fort Worth, TX 76107, USA; khadiza.zaman@unthsc.edu (K.Z.); ammarkapic@my.unthsc.edu (A.K.); vien.nguyen@unthsc.edu (V.N.)

**Keywords:** androgen deprivation, bioinformatics, canonical pathways, estradiol, gonadectomy, mass spectrometry, ocular neurodegeneration, oxidative stress, retina proteomics, retinal vulnerability, testosterone

## Abstract

Elevated oxidative stress (OS) is a primary driver of ocular neurodegeneration, worsening with age-related declines in gonadal hormones. While the loss of endogenous 17β-estradiol (E2) is a recognized risk factor for retinal degeneration in females, the impact of testosterone depletion in males remains poorly understood. To address this knowledge gap, we employed mass spectrometry-based proteomics and bioinformatic pipelines to characterize retinal protein shifts triggered by orchiectomy (ORX) in the Brown Norway rat. Proteins from ORX and intact retinas were analyzed via a discovery-driven approach using nanoflow liquid chromatography–tandem mass spectrometry with data-independent acquisition. Ingenuity Pathway Analysis^®^ of differentially expressed proteins (DEPs) revealed nearly 300 significantly regulated canonical pathways, many associated with OS, free radical detoxification, mitochondrial dysfunction and ophthalmic disease. A selected panel of DEPs was verified by protein-targeted data extraction. Notably, pathway analysis revealed the prominence of estrogen receptor signaling over androgen receptor signaling in the retina, despite the loss of male sex hormones following ORX. These findings indicate that E2-mediated pathways play a more significant role in male retinal protection than previously recognized. Our study provides the first proteomics-based evidence of the male rat retina’s heightened susceptibility to ORX-associated OS, identifying potential targets for treating sex hormone deprivation-associated retinal neurodegeneration.

## 1. Introduction

Ocular neurodegeneration, much like that affecting the brain, is a complex, poorly understood process in terms of both initiation and progression [1,2]. Currently, there is no effective pharmacological intervention to prevent, halt, or reverse neuronal damage caused by this pathological process. Many clinically relevant factors have been implicated in the diverse etiology and pathogenesis of neurodegeneration [3,4,5]. Among these, oxidative stress (OS) driven by the excessive and prolonged exposure to reactive oxygen species (ROS) that exhaust the antioxidant defense and repair systems, is one of the principal contributors [6,7,8]. Retinal neurons are particularly vulnerable to OS due to their high metabolic demand and oxygen consumption, combined with light exposure as well as a high concentration of polyunsaturated fatty acids [9]. OS has specifically been implicated in many ocular neurodegenerative diseases, including glaucoma and dry age-related macular degeneration (AMD) [10,11,12,13]. The incidence of these neuropathies increases with age, in part, due to an imbalance in or loss of redox homeostasis [14]. The resultant OS can then lead to molecular and cellular oxidative damage as well as mitochondrial and vascular dysfunctions, ultimately leading to vision impairment or even loss [15]. With aging, the endogenous production of sex hormones such as 17β-estradiol (E2) and testosterone (T) also ceases or gradually diminishes. Therefore, the association between sex hormone-induced endocrine dysregulation and the increased susceptibility to age-related neurodegenerative diseases is highly plausible [16,17,18]. Steroid hormones, such as the sex hormones, play many important non-reproductive roles throughout the central nervous system (CNS) [19,20,21]. Their de novo synthesis—including within the retina, part of the CNS—is well-established, leading them to also be classified as “neurosteroids” [22,23]. The corresponding nuclear receptors are richly, though unevenly, expressed in a sex-specific manner, influencing processes such as neuronal differentiation [24,25].

The link between the loss of the main female sex hormone E2, both natural or iatrogenic, has been extensively studied; multiple lines of research and observation corroborate that the hypoestrogenic state significantly predisposes women to neurodegenerative diseases [18,26,27]. In an ocular context, the “estrogenic” retina has been specifically identified for its role in maintaining retinal health [28,29,30]. For example, early loss of endogenous E2 has been shown to accelerate optic nerve disc aging and the development of certain glaucomatous processes [31,32]. In glaucoma, the second most common neuro-ophthalmic disorder, retinal ganglion cells (RGCs) and their optic nerve-forming axons are gradually destroyed, which over time may lead to vision loss [33]. Preclinical models overwhelmingly support the potent neuroprotective effect of E2 supplementation in ovariectomized animals [34,35,36]. This beneficial effect involves both genomic (e.g., upregulating antioxidant enzymes and neuroprotective proteins) and non-genomic signaling pathways to activate survival mechanisms [37]. Additionally, the presence of the phenolic A-ring in its structure allows for direct free radical scavenging, thus acting essentially as a chemical shield against OS [38]. Clinical observations also support the use of postmenopausal E2 replacement, within a “critical window,” to protect the retina against OS and inflammation [39,40,41]. One of the specific protective mechanisms relies on E2’s ability to activate the NRF2 (nuclear factor erythroid 2-related factor 2) pathway in the retina, subsequently triggering the production of antioxidant enzymes such as glutathione peroxidase and heme oxygenase-1; the latter also possesses potent anti-inflammatory properties [42,43].

Altogether, substantial data confirm the consensus that the loss of endogenous E2 promotes ocular neurodegeneration in females. However, a significant knowledge gap persists regarding the consequences of losing male sex hormones, specifically the primary androgen (T), in the male retina. Evidence suggests that androgens play a critical role in the pathophysiology of glaucoma, as seen in the significantly increased expression of the nuclear androgen receptors (ARs) in the glaucomatous optic nerve astrocytes in humans and monkeys [44]. Nevertheless, the literature regarding T’s specific impact remains conflicting. In animal models of AMD and Parkinson’s disease, this hormone was not found to be neuroprotective [45,46]. Conversely, other reports indicate that T exerts neuroprotective effects in the CNS directly via AR signaling rather than through its aromatization into E2 [47,48]. Clinical data are equally polarized: some studies suggest that men undergoing androgen deprivation therapy (ADT) via surgical or chemical orchiectomy (ORX) exhibit a reduced risk of developing glaucoma and AMD [49,50], while others have found that ADT leads to significant retinal thinning, a hallmark of neurodegeneration [51,52].

These contradictory findings, combined with a paucity of molecular-level research, have precluded definitive conclusions regarding the role of male sex hormone depletion in retinal/CNS health, leaving potential biomarkers and therapeutic targets undiscovered. One of our research interests focuses on how stressors disrupt the proteomic landscape and homeostatic balance. To navigate the complexity of protein regulation, state-of-the-art techniques such as mass spectrometry (MS)-based proteomics, coupled with advanced bioinformatics pipelines, have become essential for interpreting the impact of disease and treatments on protein expression, localization, and modification [53]. Therefore, the current study aimed to investigate, for the first time, the retinal proteomic shifts triggered by ORX-induced sex hormone deprivation in the Brown Norway (BN) rat, an established strain in vision research [36]. We sought to determine whether ORX alone promotes OS-associated pathways, thereby creating vulnerability to neurodegeneration in the hormone-deprived male rat retina.

## 2. Materials and Methods

### 2.1. Chemicals and Reagents

Urea, dithiothreitol, iodoacetamide, ammonium bicarbonate (ABC), and formic acid (ACS reagent grade, ≥98%) were purchased from Millipore Sigma (St. Louis, MO, USA). Sequencing-grade trypsin was from Promega (Madison, WI, USA), while Optima^®^ LC/MS-grade water, formic acid (FA), and acetonitrile (ACN) were ordered from Thermo Fisher Scientific (Waltham, MA, USA). Ketamine (100 mg/mL) and xylazine (20 mg/mL) used for euthanasia were purchased from Covetrus (Fort Worth, TX, USA).

### 2.2. Animals

The ARVO Statement for the “Use of Animals in Ophthalmic and Vision Research” served as the framework for all procedures. All protocols were approved by our Institutional Animal Care and Use Committee (approval number: IACUC-2023-0012; approved on 2 May 2023) before initiation of the studies reported here. Gonad-intact and ORX BN male rats (56–62 days old) were purchased from Charles River Laboratories (Wilmington, DE, USA). ORX surgery (*n* = 5) was performed by the supplier when the animals were 49–55 days old. The gonad-intact animals were considered the naïve, or Reference group, in the proteomics studies (*n* = 4). For euthanasia (exactly 28 days after receiving the animals), i.p. administration of ketamine (60 mg/kg body weight) and xylazine (10 mg/kg body weight) was performed. The eyes were enucleated immediately, followed by a quick dissection to collect the retina and major ocular structures, which were then stored at −80 °C until processing for proteomics studies. For the present study, one retina (randomly selected as left or right) was collected from each animal, and the contralateral retinas were reserved for separate studies.

### 2.3. Sample Preparation

Individual retinas were suspended in 200 µL of 8 M urea solution in 25 mM ABC for 1 h, followed by centrifugation to collect the supernatant, as previously described [54,55]. Protein content was measured using a BioTek Synergy H1 microplate reader with a Take3 microplate (Agilent, Palo Alto, CA, USA). Samples were normalized to 100 µg of protein each, reduced with dithiothreitol, and carbamidomethylated with 5 mM of iodoacetamide at room temperature in the dark, according to standard protocols [54,55]. After diluting the samples with 25 mM ABC to reduce the urea concentration below 2 M, proteins were digested overnight at 37 °C with 2 μg trypsin per sample. Digestion was quenched by adding 5 µL of formic acid (FA). Next, individual samples were cleaned up on Sep-Pak™ C-18 cartridges (Waters, Milford, MA, USA) and dried in a Vacufuge™ vacuum centrifuge concentrator (Eppendorf AG, Hamburg, Germany). The residues were stored at −80 °C until analysis.

### 2.4. Nanoflow Liquid Chromatography–Tandem Mass Spectrometry

The dried samples were reconstituted in 5% (*v*/*v*) aqueous ACN containing 0.1% (*v*/*v*) FA to produce 1 µg/µL protein-equivalent concentration. Data were collected by data-independent acquisition (DIA) on an Orbitrap Fusion Tribrid instrument equipped with a Nanospray Flex™ source and coupled to an EASY nLC-1200 system (Thermo Fisher Scientific, San Jose, CA, USA), as described previously [56]. Briefly, 5 µL of sample aliquots were injected onto a Thermo Fisher Scientific Acclaim™ PepMap™ 100 octadecylsilica (C18) trap column (2 cm × 75 μm i.d., packing particle size of 3 µm) at 1 µL/min, followed by elution and reversed-phase separation on an Acclaim™ PepMap™ 100 C18 column packed (15 cm × 75 μm i.d., packing particle size of 2 µm) at 300 nL/min using a 45 min gradient program [56]. Full-scan mass spectra were obtained at a nominal mass resolution of 120,000 (defined at *m*/*z* 200) in the Orbitrap with the automatic gain control (AGC) target set to 400,000. Over the precursor scan range of *m*/*z* 385 to 1015, DIA scans were acquired with *m*/*z* 24 isolation windows and higher-energy collisional dissociation fragmentation performed at a 33% normalized collision energy, and the Orbitrap’s AGC target was adjusted to 50,000 for the detection of the product ions.

### 2.5. Data Analysis

First, Spectronaut (Version 18; Biognosys, Zurich, Switzerland), relying on the UniProtKB *Rattus norvegicus* protein database (species: *Rattus norvegicus*, 2023; 36,206 entries), was used to process the raw collected files, assuming trypsin as the digestion enzyme (with a maximum of one missed cleavage) and carbamidomethylation of cysteine as a fixed modification. A false discovery rate (FDR) of <0.01 (1%) was applied to the Pulsar search algorithm’s directDIA + (Deep) workflow for peptide-spectrum matches, peptide FDR, and protein group FDR. Quantitative values for the identified proteins were obtained by label-free quantitation (LFQ) using Spectronaut’s built-in intensity-based fragment-ion selection strategy. Statistically significant differences were determined from the protein expression profiles using Spectronaut’s internal algorithm relying on unpaired *t*-tests (*p* < 0.05) and Benjamini–Hochberg (BH) correction for multiple testing [56].

A follow-up processing of the raw data files, focused on a panel of selected proteins, was conducted using ProteoWizard (version 3.0.19254) integrated into the Scaffold DIA (version 3.2.1, Proteome Software, Portland, OR, USA). After conversion to the mzML format and retention time alignment, a spectral library search was performed against Rat_prosit_generated_library.dlib with a peptide mass tolerance of 20 ppm and a fragment mass tolerance of 10 ppm. Search criteria included trypsin as the digestion enzyme (allowing a maximum of one missed cleavage), carbamidomethylation of cysteine as a fixed modification, and the inclusion of peptides with charges (+) of 2 to 5 and lengths of 6 to 30 amino acid residues. Identified peptides were filtered using Scaffold DIA’s integrated Percolator (version 3.01) to maintain an FDR of ≤0.01 (1%). Peptide quantification was performed using the software’s implementation of EncyclopeDIA (version 1.12.31), where the five highest-quality fragment ions per peptide were selected for quantification [56]. Finally, unpaired *t*-tests were performed using the Scaffold DIA’s built-in statistics module to identify statistically significant differences in the protein abundances (DEPs) between the groups (*p* < 0.05).

### 2.6. Bioinformatics

Proteins with statistically significant differences in expression (DEPs) from two-group comparisons were submitted to Ingenuity Pathway Analysis^®^ (IPA^®^, Redwood City, CA, USA) to link them with canonical pathways, diseases, and functions, as well as protein–protein interaction networks. Overlaps of *p*-values were reported from IPA^®^’s calculations using the right-tailed Fisher’s exact test. Z-scores, which assign directionality to a function or pathway (orange for activation/increase, blue for inhibition/decrease, and “0” for no change) as well as predicted signaling patterns, were generated using the Molecule Activity Predictor (MAP) tool of IPA^®^ Knowledge Base.

## 3. Results

### 3.1. Shotgun Proteomics to Reveal the Effect of Orchiectomy on the Male Rat Retina’s Proteome

**Figure 1:**
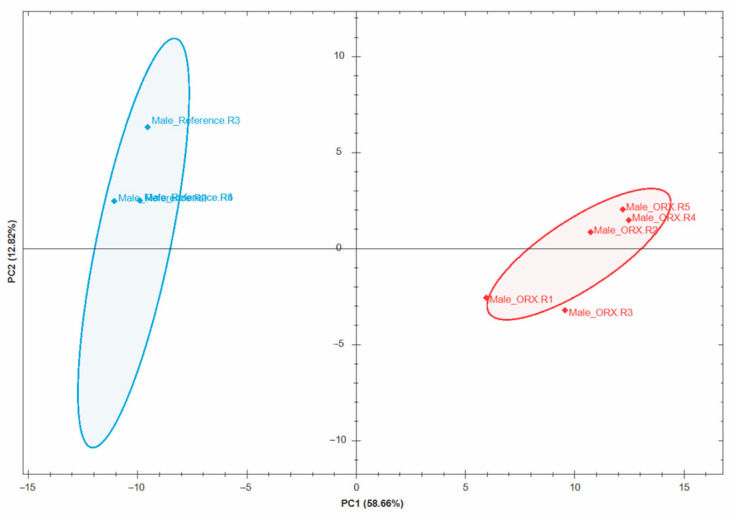
PCA plot showing the dissimilarity between the Reference (intact, blue) and ORX (red) male BN rat retinas.

Analysis of the DIA dataset by Spectronaut identified over 3200 high-confidence proteins at 1% FDR. From these, more than 700 differentially expressed proteins (DEPs) were found between the Reference (naïve, intact) and ORX retinas based on LFQ and unpaired *t*-tests with multiple testing using BH correction. Specifically, 386 proteins were downregulated, and 349 proteins were upregulated following sex hormone deprivation (Table S1). A graphical representation of these findings by principal component analysis (PCA) using Spectronaut software is displayed in Figure 1.

**Figure 2:**
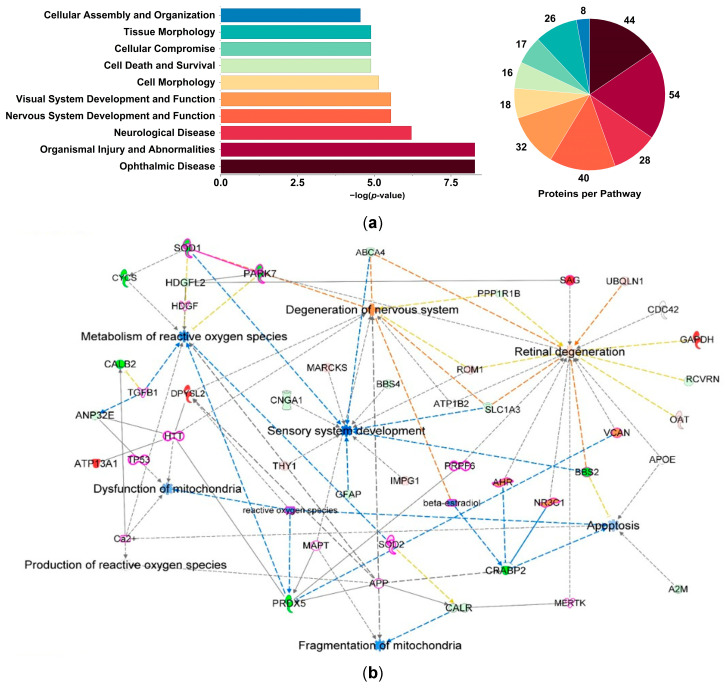
ORX-impacted proteins mapped by IPA^®^ and linked to OS response in the male BN rat retina. (**a**) Bar chart showing the top diseases and physiological functions represented by DEPs between the ORX and the Reference groups. The pie chart shows the number of proteins per pathway. (**b**) IPA^®^ Knowledge Base linked a subset of DEPs to disease and function networks associated with ROS production, metabolism of ROS, mitochondrial dysfunction, mitochondrial fragmentation, sensory system development, nervous system degeneration, retinal degeneration, and apoptosis. Blue dashed line: inhibition/decrease; orange dashed line: activation/increase; yellow dashed line: cannot be predicted; blue solid line: inhibition. Purple solid line: protein interaction network merged using IPA^®^ pathway explorer; purple borders: intermediary key proteins connecting the subset of DEPs to diseases and functions. Gray solid/dashed lines: direct/indirect relationship; red: upregulation; green: downregulation. The shade of color is indicative of the extent of change in protein expression; solid line: direct relationship; dashed line: indirect relationship. Abbreviations of proteins and overlaid functions are listed in Table S5a.

The IPA^®^’s curated Knowledge Base associated these DEPs with nearly 40 disease-, molecular-, and physiological function-related pathways (Table S2), in addition to more than 300 canonical pathways (Table S3a), and assembled 13 significant protein interaction networks (Table S4). In Figure 2, we summarized the top disease- and function-related events triggered by ORX, focusing on OS in the context of ocular neurodegeneration. Specifically, Figure 2a includes the top 10 diseases and functions modulated by ORX (sex hormone deprivation), with ophthalmic disease being one of the topmost regulated categories (left panel), accompanied by a pie chart representing the number of pathway-associated proteins (right panel). Additionally, Figure 2b displays DEPs involved in different aspects of OS and ocular neuropathies, including retinal and photoreceptor degeneration, neurodegeneration, and synaptic transmission. The MAP tool of IPA^®^ predicted intermolecular outcomes of protein interactions and assigned directionality to the pathways or biological functions. From this figure, the adverse effects of ORX alone are evident, including predicted decreases (or inactivation) of ROS metabolism, mitochondrial fragmentation, and apoptosis, as well as disruption of the sensory system that contributes to nervous system activation and retinal degeneration. In the present context, Figure 2b also shows markers of OS induced by ORX, such as superoxide dismutase (SOD1), Parkinson disease protein homolog (PARK7), and peroxiredoxin 5 (PRDX5).

**Figure 3:**
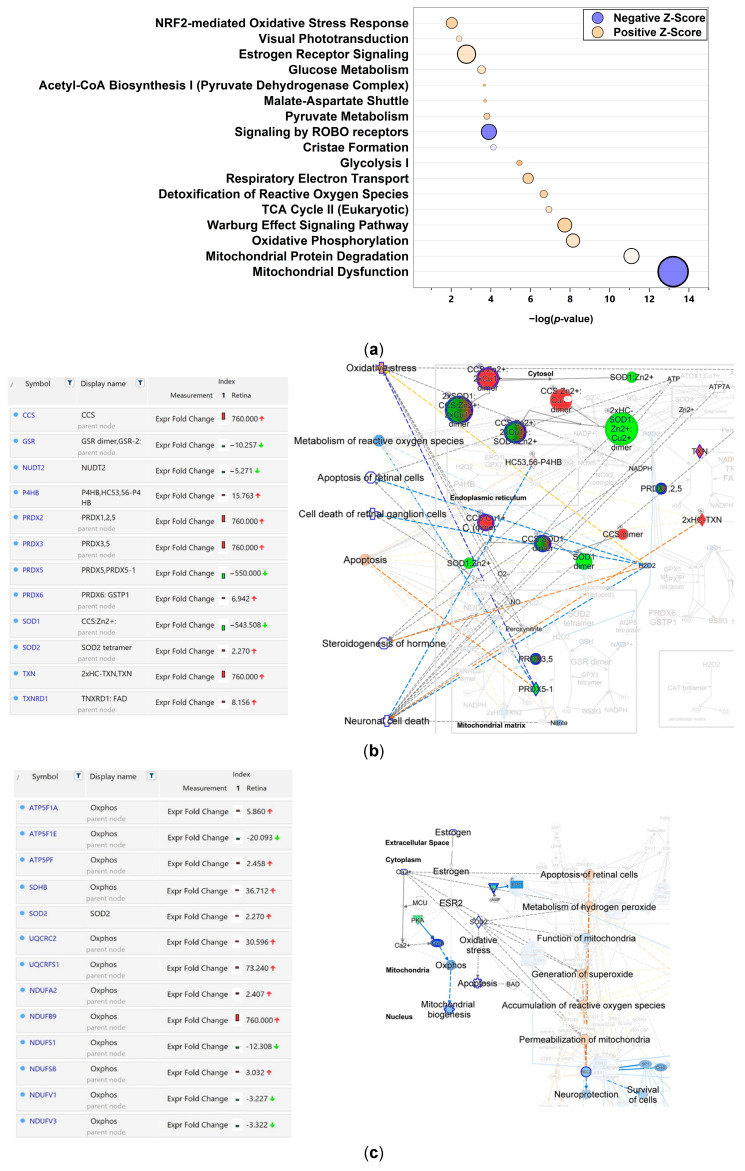
ORX-impacted proteins involved in canonical pathways linked to OS response in the male BN rat retina. (**a**) Bubble chart showing the topmost canonical pathways associated with OS response. The size of bubbles indicates more proteins overlapping with that pathway. Orange and blue colors represent predicted activation and suppression, respectively. (**b**) Focused view on the detoxification of the ROS with regulated proteins (left side panel), illustrating the direct and indirect relationships with various aspects of OS linked to neurodegeneration. Protein names abbreviated in this figure with their functions are given in Table S5b. (**c**) Focused view on the ESR signaling pathway, emphasizing OS-related components and regulated proteins (left side panel). A list of all protein names abbreviated in this figure with their overlaid functions is given in Table S5c. In (**b**,**c**), blue dashed line: inhibition/decrease; orange dashed line: activation/increase; yellow dashed line: findings cannot be predicted; gray lines: relationship between nodes (solid: direct; dashed: indirect). The shade of color is indicative of the extent of change in protein expression; red: upregulation; green: downregulation.

IPA^®^ also identified over 70 regulated canonical pathways (Table S3b) linked to DEPs in the retina following sex hormone deprivation. Figure 3a shows a bubble chart of the top canonical pathways associated specifically with responses to OS. These associations included mitochondrial dysfunction and ROS detoxification, as well as OS response mediated by NRF2. Other pathways involved metabolism and bioenergetics, such as oxidative phosphorylation (OXPHOS), Warburg effect signaling, and glycolysis. Along with estrogen receptor (ESR) signaling, visual phototransduction, and ROBO receptor signaling, these were among the top IPA^®^ canonical pathways associated with DEPs in the retina following ORX.

Using IPA^®^ Focused View feature, Figure 3b,c displays segments of two highly relevant canonical pathways taken from the list in Figure 3a. Specifically, Figure 3b (full view shown in Figure S1) illustrates the ROS detoxification pathway and demonstrates the impact of sex hormone deprivation on numerous proteins, such as peroxiredoxins (PRDX-1, 2, 3, 5, 6), superoxide dismutase (SOD1/SOD2), thioredoxin (TXN), glutathione reductase (GSR), and protein disulfide isomerase (P4HB). Their fold changes, determined from our DIA proteomics dataset, are shown on the left, while their molecular interplays are shown in the interaction network on the right. To translate these findings into biological outcomes, we overlaid disease and function categories; these indicated decreases in ROS metabolism, retinal degeneration, apoptosis, steroid hormone synthesis, and neuronal cell death as associated processes. Since ESR was among the top canonical pathways (Figure 3a), we also analyzed this signaling pathway, focusing on the segment that addresses OS and the inhibition of mitochondrial biogenesis (Figure 3c; full view in Figure S2). This focused view (scheme on the right) revealed many critical aspects of neurodegeneration, including increased OS and mitochondrial permeabilization. The left panel of Figure 3c displays the regulatory patterns of many proteins involved in OXPHOS and the OS response leading to neurodegeneration, such as cytochrome b-c1 complex subunit 2 (UQCRC2), NADH dehydrogenase (ubiquinone) 1 alpha subcomplex subunit 2 (NDUFA2), and succinate dehydrogenase subunit 8 (SDH8).

### 3.2. Identification of Potential Markers of Orchiectomy-Trigged Oxidative Stress in the Male Rat Retina

**Figure 4:**
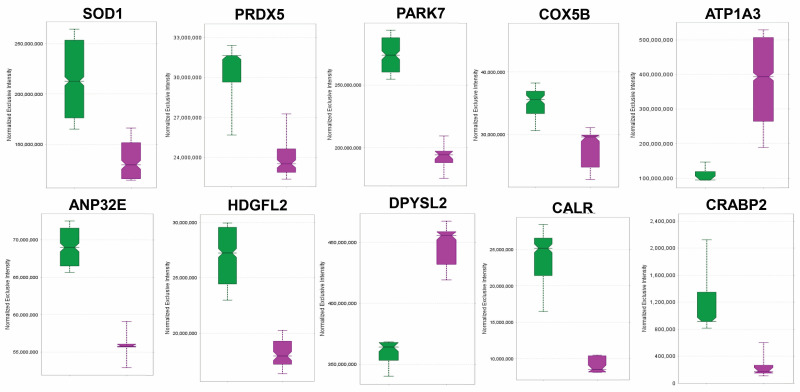
Scaffold DIA-based quantitative survey measuring the impact of ORX on the selected panel of OS-associated DEPs in the male BN rat retina. Box plots were generated by the software: Reference—green boxes and ORX—magenta boxes. All DEPs showed statistically significant differences (unpaired *t*-tests by Scaffold DIA’s built-in statistics module; *p* < 0.05, *n* = 4–5). Abbreviations of protein names are listed in Table S6a.

Based on our analysis summarized in Figure 2 and Figure 3, we selected a panel of DEPs (Table S6a) as potential retinal markers of ORX-trigged OS. In Figure 4, we show the sex hormone deprivation-associated patterns of the selected DEPs using Scaffold DIA analysis. Transitions used for their LFQ are listed in Table S6b. We displayed the impact of ORX (magenta boxes) on these OS-associated protein makers by comparing their expression levels with those in the Reference group (intact animals, green boxes), which clearly indicated dysregulation of these retinal proteins following ORX.

**Figure 5:**
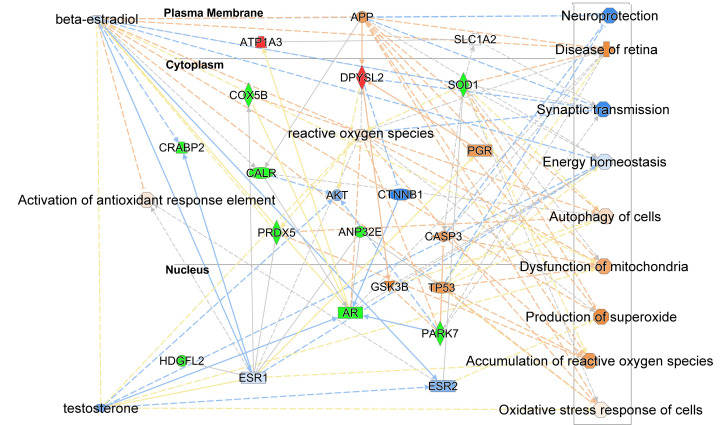
Interaction network generated by IPA^®^ Pathway Explorer among the selected DEPs (CRABP2, CALR, HDGFL2, PARK7, ATP1A3, ANP32E, COX5B, PRDX5, SOD1, and DPYSL2), E2, T, and their nuclear receptors (ESRs and AR, respectively), as well as elements of OS responses. Blue dashed line: inhibition/decrease; orange dashed line: activation/increase; yellow dashed line: cannot be predicted; blue solid line: inhibition; gray solid/dashed lines: direct/indirect relationship; red: upregulation; and green: downregulation. The shade of color is indicative of the extent of change in protein expression. Solid line: direct relationship; dashed line: indirect relationship. Abbreviations of protein names are listed in Table S5d.

Although AR signaling was not included among the topmost canonical pathways associated with OS response (Figure 3a), we nevertheless explored the direct and indirect links between these DEPs and hormone loss in the retina following ORX. Therefore, both T and E2, their nuclear receptors [AR, ESR1 (ESRα), ESR2 (ERβ)], as well as elements associated with OS responses, were included in this exploration. By combining machine learning techniques with other heuristics, the IPA^®^ Pathway Explorer tool created a network that enabled us to visualize the complex links among these components (Figure 5), involving the expected deprivation of T and E2 (Figure 5) and their association with the network predicted by the IPA^®’^ knowledge. The interaction network depicted in this figure also shows that E2 directly activates (orange dashed line) the antioxidant response element (ARE); however, there is no such pathway for T.

To provide potential pathophysiological context, we overlaid the network with disease and function categories to visualize the associations of the selected DEPs with OS, as well as cellular and neuronal functions. The MAP tool in IPA^®^ projected an increase in superoxide production, accumulation of ROS, activation of the OS mechanism, and mitochondrial dysfunction. These findings ultimately point toward increased retinal disease and a decrease in synaptic transmission and energy homeostasis, as well as potentially diminished neuroprotection (Figure 5, side panel on the right), leading to increased vulnerability to the sex hormone deprivation-induced neurodegeneration and retinal disease.

The network shows that AR directly mediates the regulation of cytochrome C oxidase 5B (COX5B), PARK7, calreticulin (CALR), Na^+^/K^+^ ATPase alpha-3 subunit (ATP1A3), the acidic nuclear phosphoprotein 32 family member E (ANP32E), and dihydropyrimidinase-related protein 2 (DPYSL2). We can also recognize the increase or activation of ROS and the OS response based on the predicted decrease in T and its nuclear receptor following sex hormone deprivation. On the other hand, E2 itself, as well as in conjunction with ESRs and AR, is linked to the regulation of cellular retinoic acid-binding protein 2 (CRABP2), CALR, PRDX5, SOD1, ANP32E, and hepatoma-derived growth factor-related protein 2 (HDGFL2). The deprivation of E2 due to ORX is predicted to be associated with increased ROS levels, triggering OS responses. In contrast to T, IPA^®^ implicated a definitive OS response mediated by E2. Additionally, downregulation of E2 and ESRs is predicted to trigger activation of antioxidant response elements (AREs).

## 4. Discussion

This study aimed to investigate, for the first time, the impact of ORX on the BN rat retinal proteome. We hypothesized that sex hormone deprivation creates an environment that potentially increases retinal vulnerability to neurodegeneration, similar to the processes proposed for the hypoestrogenic, aging retina in females [18,26,27,28,29,30]. Our discovery-driven shotgun proteomics approach employed DIA, a highly reproducible and accurate method that provides high protein coverage [56]. The PCA plot in Figure 1 illustrates that the retinal proteomes of the ORX and Reference (naïve, intact) groups were markedly different, as shown by the lack of overlap. More than 700 proteins were identified as DEPs in response to ORX (Table S1), and IPA^®^ linked them to several diseases and physiological functions (Table S2). The top identified areas included ophthalmic and neurological diseases, organismal injury and abnormalities, and visual and nervous system development (Figure 2a). The enrichment of visual and nervous system pathways confirms that many of the DEPs are involved in retinal neurons, despite the unbiased nature of our protein list.

IPA^®^ also generated over 300 canonical pathways (Table S3a) and 13 significant protein interaction networks (Table S4). Here, however, we focused on canonical pathways associated with OS response triggered by sex hormone deprivation (Table S3b). According to the protein interaction network based on disease and physiological functions (Figure 2b), neurodegeneration of the CNS, mitochondrial dysfunction and fragmentation, apoptosis, as well as ROS metabolism, were among the most affected pathways in the ORX retina. Many prominent antioxidant-related proteins were also dysregulated in this network, suggesting that ORX impacted the regulation of retinal proteins vital for managing ROS and neuronal functions. In addition, the expressions of a subset of proteins associated with photoreceptors, including recoverin (RCVRN) and cyclic nucleotide-gated channel subunit alpha 1 (CNGA1), were reduced in the ORX animals (Figure 2b, Tables S2 and S3a). While both RCVRN and CNGA1 are used as markers for photoreceptors and are involved in phototransduction, their expression in bipolar cells has also been reported [57]. Notably, the gradual suppression of CNGA1 by antisense mRNA alone has been shown to induce apoptosis in photoreceptors and, interestingly, in bipolar cells. Reduced expression of these proteins potentially suggests reduced photoreceptor function in the ORX animals.

The detoxification of the ROS pathway identified in Figure 3a was further analyzed through a focused-view network (Figure 3b, full view shown in Figure S1). The downregulation of PRDX5 and SOD1 suggests that ROS metabolism is significantly reduced in the ORX retina. Removal of the sex hormones also negatively affected other antioxidant proteins, leading to the downregulation of glutathione reductase (GSR) and the upregulation of PRDX 3,4,6, as well as SOD2, TXN, and thioredoxin reductase 1 (TXNRD1). Interestingly, ORX also triggered a rise in copper chaperone for SOD1 (CCS). Although CCS typically stabilizes SOD1, its elevation may be a compensatory response to sex hormone deprivation, as its overexpression has been reported to exacerbate neurodegeneration [58]. Most notably, we observed an inverse relationship where CCS was upregulated while its target, SOD1, was downregulated (Figure 2b and Figure 3b). We are unaware of previous reports on this inverse expression pattern between SOD1 and CCS in the context of sex hormone deprivation. Overall, this downregulation of antioxidant proteins may indicate vulnerability to OS, as the retina is among the most metabolically active tissues and is exposed to ROS-producing UVB radiation [11].

Surprisingly, in the bubble chart in Figure 3a, one of the most highly enriched canonical pathways is ESR signaling—thus, mediated by the nuclear E2 receptors—rather than T-mediated AR signaling (Figure S3), despite the loss of male sex hormones via ORX (ESR signaling *p* = 1.35 × 10^−4^, 30 proteins; AR signaling *p* = 2.36 × 10^−2^, 12 proteins). Additionally, IPA^®^ MAP predicted inhibited ROS metabolism and mitochondrial dysfunction in the ORX retina (Figure 2b, Table S3). Therefore, another focused view on the ESR signaling pathway, centering on mitochondrial activity, was explored (Figure 3c, full view is given in Figure S2).

Thus, in Figure 3c, we see the downregulation of OXPHOS, which may be related to impaired mitochondrial function and increased ROS [59]. That is consistent with the content in Figure 2b regarding the reduced expression of CYCS (cytochrome C, somatic) by ORX. While CYCS negatively regulates ferroptosis when complexed with inositol polyphosphate-4-phosphatase type [60], in our study, we found that ferroptosis, iron uptake, and transport were enriched pathways in the ORX retina (Table S3a). Dysregulations of iron homeostasis and ferroptosis have been associated with ocular diseases, including light-induced photoreceptor degeneration, AMD, and glaucoma [61]. Although CYCS is primarily known as an electron carrier that shuttles electrons from cytochrome b-c1 (Complex III), we observed, rather unexpectedly, the upregulation of the Complex III subunits UQCRC2 and UQCRFS1 (Figure 3c). While these subunits are involved in the stabilization and formation of Complex III, the concurrent reduction in CYCS may suggest the existence of other, non-canonical functions [62,63]. Additionally, the expression of the other respiratory complexes exhibits an intricate pattern of dysregulations; specifically, several subunits of the NADH dehydrogenase complex (Complex I), including NDUFA2, NDUFB9, NDUFS1, NDUFS8, NDUFV1, and NDUFV3, showed altered protein expression levels. The ATP synthase (ATPS, Complex V) F_0_ and F_1_ subunits were also significantly impacted by ORX; ATP5F1A and ATPSPF were upregulated, while ATP5F1E was downregulated (Figure 3c). The shift in the expression levels of these proteins—which are critical for the formation, elimination, and monitoring of ROS—may lead to electron leakage, likely contributing to OS [64]. Altogether, we found dysregulation across Complexes I, III, and V, along with the unexpected reduction of CYCS, which potentially signifies a significant breakdown in mitochondrial bioenergetics following ORX.

Next, we conducted a quantitative survey (Figure 4, Table S6) of selected DEPs, representing a panel of potential markers in the ORX BN rat retina (Figure 2b and Figure 3b,c). These proteins are associated with many critical functions in retina health, including antioxidant defense and mitochondrial function, as well as neuronal regulation, in the retina. Among the antioxidant proteins, SOD1 was downregulated in ORX animals (Figure 4). This protein was specifically associated with the predicted reduction of ROS metabolism and increased nervous system degeneration and was linked to several neurodegenerative canonical pathways (Figure 2b and Table S3a,b). However, growing evidence suggests that SOD1 can also regulate OS-associated proteins and metabolic processes, such as aerobic glycolysis [65,66]. In RGCs, SOD1 was specifically found to be protective against OS induced by N-methyl-d-aspartate-mediated excitotoxicity [67]. Another downregulated protein is PRDX5, an atypical mitochondrial peroxiredoxin that exhibits antioxidant activity through enzymatic reactions with ROS and reactive nitrogen species. Previous studies have reported its expression in RGCs, photoreceptors, and amacrine cells, as well as in the optic nerve [68]. Other functions of PRDX5 include providing neuronal resistance to stress; its overexpression protects neurons from ROS and cellular death in various animal models, whereas silencing PRDX5 increases vulnerability to OS [69]. Thus, based on the reduced PRDX5 expression in our dataset, ORX may promote retinal vulnerability to OS by the downregulation of this protein (Figure 4).

PARK7, which acts as a ROS sensor and a mediator of OS response [70], was also reduced by ORX (Figure 2b and Figure 4). This protein acts as a modulator of NRF2 [71], which regulates many of the previously listed proteins, such as SOD1, in response to OS (Figure 2b and Figure 3b,c). Therefore, reduced PARK7 levels may imply a diminished cellular capacity for OS monitoring and response. In addition to acting as a ROS sensor, the protein also possesses several auxiliary functions, including the prevention of neurotoxic Lewy body formation by inhibiting α-synuclein aggregation [72]. While Lewy bodies are predominantly studied in the context of dementia and Parkinson’s disease in the brain, they have also been observed in the retina and optic nerves of patients with these conditions, potentially through retrograde transsynaptic dysfunction [73]. Thus, the loss of PARK7 may promote the formation of neurotoxic aggregations in the vulnerable retina. In the midbrain astrocytes of PARK7 knockout mice, mRNA expression of NRF2 and cytochrome P450 1B1 (which mediates sex steroid and retinoic acid metabolism [71]) was downregulated in the male animals, suggesting greater sensitivity of males to the loss of PARK7 and OS; however, it is unclear whether this pattern also exists in the retina.

COX5B is a vital nuclear-encoded component of the mitochondrial electron transport chain and is one of the many subunits that make up Complex IV [74]. In this study, the expression of COX5B was downregulated in the ORX group (Figure 4). This repression may impair mitochondrial energy production by altering the structure or function of the complex. While little specific information on the retina has been reported, COX5B is known to be suppressed in individuals with neurodegenerative diseases, such as Alzheimer’s disease, and in corresponding animal models; this suppression potentially contributes to reduced ATP synthesis in neurons [75]. This protein has also been shown to interact with the AR, suggesting a mechanism in which the AR regulates the trafficking of COX5B into the mitochondria [76]. Furthermore, studies using cancer cell models show that elevated COX5B expression is associated with pro-survival signaling and enhanced mitochondrial function [77]. Therefore, the ORX animals may have compromised mitochondrial bioenergetics compared to the intact animals (Reference group).

In the retina, ATP1A3 is expressed solely in retinal neurons, with most studies focusing on its role in photoreceptors [78]. We found elevated ATP1A3 levels in the retinas of ORX animals. Notably, mutations resulting in the overexpression of ATP1A3 cause optic nerve atrophy and may be associated with rod-cone dystrophy [78]. Figure 4 also shows that ORX reduced the expression of ANP32E and HDGFL2, both of which are understudied in the retina. While their transcription has been reported in developing retinas, their specific cell-type distribution remains unclear. ANP32E is a histone chaperone associated with chromatin remodeling and transcriptional regulation [79]. This protein also forms a complex with cerebellar protein developmental regulated 1 (CPD1) and is involved in cerebellar synaptogenesis [80]. However, as no studies have examined its effects on the retina, we are the first to report that ANP32E is affected by ORX in the BN male rats, suggesting it may play a role in neurodegenerative processes (Figure 2b).

Although HDGFL2 is expressed in neurons [81], little research has examined its role in retinal neurons. While expressions of HDGF (hepatoma-derived growth factor) and HGDFL3 (hepatoma-derived growth factor-related protein 3) have been reported in the nuclei of RGCs and photoreceptors as angiogenic promoters, HGDFL2 has not yet been characterized [82]. Its primary functions include involvement in DNA repair mechanisms and epigenetic regulation [83]. Proteomic analysis of HDGDL2 immunoprecipitation experiments in cultured neuronal cells reveals interactions with ribosomes and transcription-associated mechanisms [84]. However, we are the first to report that HDGFL2 is potentially regulated by sex hormones in the retina. Therefore, a reduced expression following ORX may increase vulnerability to OS-mediated DNA damage or affect the expression of pro-survival proteins; however, this requires further evaluation. Reduced expression of both ANPE32E and HDGFL2 (Figure 4) could also contribute to elevated sensitivity to retinal degeneration.

DPSYL2, also known as CRMP-2, is a microtubule-associated protein involved in regulating axonal guidance, synaptic function, and synaptogenesis during and after CNS development [85,86]. Most studies involving DPYSL2 have revealed relationships with neurological dysfunctions [87]. In addition to its role in early retinal differentiation, DPYSL2 was identified via proteomic analysis of retina-conditioned media as potentially involved in stem cell differentiation into neural retina precursor cells [88]. Interestingly, suppressed phosphorylated DPSYL2 was neuroprotective against RGC loss in an optic nerve crush model of retinal neurodegeneration [89]. Therefore, measuring phosphorylated DPSYL2 may provide further insight into its altered expression pattern in ORX animals. According to the IPA^®^ Knowledge Base, the upregulation of DPYSL2 is indirectly associated with ROS metabolism and neurodegeneration (Figure 2b and Figure 4).

CALR is a multifunctional endoplasmic reticulum (ER) chaperone protein involved in protein quality control. It binds misfolded ER proteins, preventing their export to the Golgi apparatus, while also regulating intracellular calcium [90]. For these reasons, CALR is often used as a histological marker of ER localization. However, under stress, CALR additionally plays a role in preventing the unfolded protein response and maintaining mitochondrial function [91]. In the retina, CALR can translocate to the rod surface and act as a damage-associated molecular pattern [92]. Our study found that CALR was associated with several pathways, including cyclophilin, AR, and ER stress signaling (Table S3a). Potentially, the dysregulation of cyclophilin signaling pathways may skew retinal microglia toward proinflammatory phenotypes; however, this remains to be confirmed. Another potential mechanism by which CALR could affect intracellular calcium levels is its role in stress resistance, as shown in one study that found that RGCs with elevated calcium levels were resistant to death after optic nerve crush [93]. While CALR was reduced in the ORX animals (Figure 2b and Figure 4), analyzing the translocation of the protein may reveal more information regarding the physiological state of the retina.

Expression of CRABP2, which is heavily involved in the retinoic acid signaling pathways [94], was also reduced in the ORX animals (Figure 2b and Figure 4, as well as Table S3a). Upon binding all-trans retinoic acid (ATRA), CRABP2 can translocate to the nucleus to activate retinoic acid receptors (RARs)—including RARA, RARB, RARG, and RXRs—or deliver ATRA for catabolic processes via cytochrome proteins. CRABP2 expression specifically has been shown to affect the transcription rate of RAR genes, at least in cancer models. Therefore, RAR activation in ORX animals may be reduced due to the repressed CRABP2 expression (Figure 2b). RAR-mediated protein expression includes those associated with neuronal development and regeneration. While further studies are needed to isolate the impact of CRABP2 in the ORX male rat retina, there is a potential reduction in regeneration capacity following ORX. Furthermore, RAR signaling is vital for maintaining neuronal function and activity [95]. To our knowledge, this is the first report on these proteins in the male retina under sex hormone deprivation.

Among the many pathways associated with the identified DEPs (Figure 2b and Figure 3b,c and Tables S2 and S3), ESR was among the most highly represented. While T is considered the primary male sex hormone, intracellular aromatase converts it to E2. Both aromatase and the ESR are expressed in the retina of male rats [22]. Therefore, we explored the relationship between the sex hormone receptors (AR and ESRs) and our sex hormone deprivation-associated protein panel (Figure 4) using various heuristic bioinformatic approaches in IPA^®^ (Figure 5). Generally, ORX phenotypes were characterized by reduced neuronal function, elevated OS, and potentially increased retinal vulnerability. As expected, both T and E2 were predicted to be downregulated based on the protein expression pattern of our panel (Figure 5). Interestingly, T, through AR activity, interacts with only a few proteins in our panel (Figure 4) and is not directly associated with regulating OS, as it does not interact with AREs. These AREs act as a switch to activate antioxidant defense mechanisms in response to OS [96]. According to IPA^®^ Knowledge Base, this observation highlights a significant difference in how T and E2 regulate the response to OS.

## 5. Conclusions

In summary, we utilized MS-based proteomics to provide an in-depth analysis of the effect of ORX in the BN male rat retina. Our DIA-based, discovery-driven protein informatics revealed that ORX profoundly promotes OS, as evidenced by the cascade of DEP-enriched pathways, disease states, and physiological functions linked to the OS response. For example, the inverse relationship between CCS and SOD1, alongside the dysregulation of SOD1, PRDX5, and PARK7, suggests an impaired antioxidant defense and increased vulnerability to retinal neurodegeneration. Pathway analyses further suggest that ESR signaling predominates over AR signaling in the ORX retina, despite the loss of male sex hormones. Our study offers the first proteomics-based evidence of the male rat retina’s heightened susceptibility to ORX-associated OS, identifying potential targets for treating retinal neurodegeneration associated with sex hormone deprivation.

## Data Availability

The mass spectrometry proteomics data have been deposited to the ProteomeXchange Consortium via the PRIDE [97] partner repository with the dataset identifier PXD075145.

## Supplementary Materials

The following supporting information can be downloaded at: https://www.mdpi.com/article/10.3390/antiox15040479/s1. Figure S1: Full view of detoxification of the ROS pathway illustrated in Figure 3b. Figure S2: Full view of ESR signaling pathway illustrated in Figure 3c. Figure S3: Full view of the AR signaling pathway. Table S1: Differentially expressed proteins (DEPs) comparing the retinas of ORX and Reference groups. Proteins that met the fold change cutoffs ≥1.5 or −1.5 and were statistically significant based on *t*-test with BH correction. Table S2: Disease, molecular, and functional pathways generated by IPA^®^ are based on the submitted DEPs comparing the retinas of the ORX and Reference groups. Table S3a: IPA^®^-generated canonical pathways based on the submitted DEPs comparing the retinas of ORX and Reference groups. Table S3b: IPA^®^-generated canonical pathways related to OS based on the submitted DEPs comparing the retinas from ORX and Reference groups; Table S4: List of all protein interaction networks generated from the DEPs between the ORX and Reference rat retinas; Table S5a: List of abbreviations and overlaid functions shown in Figure 2b. Table S5b: List of abbreviations of proteins shown in Figure 3b. Table S5c: List of abbreviations and overlaid functions of proteins shown in Figure 3c. Table S5d: List of abbreviations and overlaid functions of proteins shown in Figure 5. Table S6a: Scaffold DIA-based quantitative survey measuring the impact of ORX on the selected panel of OS-associated DEPs in the male rat retina. Table S6b: Transitions used for the quantitation of DEPs shown in Figure 4.

## Abbreviations

The following abbreviations are used in this manuscript:

ABC	Ammonium bicarbonate
ACN	Acetonitrile
ADT	Androgen deprivation therapy
AGC	Automatic gain control
AMD	Age-related macular degeneration
ANP32E	Acidic nuclear phosphoprotein 32 family member E
AR	Androgen receptor
ARE	Antioxidant response element
ATP1A3	Na^+^/K^+^ ATPase alpha-3 subunit
BN	Brown Norway
CALR	Calreticulin
CCS	Copper chaperone for superoxide dismutase
CNGA1	Cyclic nucleotide-gated channel subunit alpha 1
CNS	Central nervous system
COX5B	Cytochrome C oxidase 5B
CPD1	Cerebellar protein developmental regulated 1
CRABP2	Cellular retinoic acid-binding protein 2
CYCS	Cytochrome C, somatic
DEP	Differentially expressed protein
DIA	Data independent acquisition
DPYSL	Dihydropyrimidinase-related protein
E2	17β-Estradiol
ESR	Estrogen receptor
FA	Formic acid
FDR	False discovery rate
GSR	Glutathione reductase
HDGFL	Hepatoma-derived growth factor-related protein
IPA^®^	Ingenuity Passway Analysis^®^
LC	Liquid chromatography
LFQ	Label-free quantitation
MAP	Molecule activity predictor
MS	Mass spectrometry
MS/MS	Tandem mass spectrometry
NDUFA2	NADH dehydrogenase (ubiquinone) 1 alpha subcomplex subunit 2
NRF2	Nuclear factor erythroid 2-related factor 2
ORX	Orchiectomy
OS	Oxidative stress
OXPHOS	Oxidative phosphorylation
P4HB	Protein disulfide isomerase
PARK7	Parkinson disease protein homolog
PRDX	Peroxiredoxin
RAR	Retinoic acid receptor
ROS	Reactive oxygen species
RGC	Retinal ganglion cell
RCVRN	Recoverin
SDH8	Succinate dehydrogenase subunit 8
SOD	Superoxide dismutase
T	Testosterone
TXN	thioredoxin
TXNRD1	Thioredoxin reductase 1
UQCRC2	Cytochrome b-c1 complex subunit 2
UQCRFS1	Ubiquinol-cytochrome c reductase
